# Sustaining the community dispensation strategy of HIV antiretroviral through community participation

**DOI:** 10.1186/s40249-019-0518-8

**Published:** 2019-01-24

**Authors:** Bibiane Siaheu Kameni, Jobert Richie Nansseu, Sandra Ayuk Tatah, Jean Joel Bigna

**Affiliations:** 1 HIV day care Unit, Ngaoundéré Regional Hospital and Adamawa Regional Technical Group for the fight against HIV/AIDS, Ngaoundéré, Cameroon; 2 Department of Public Health, Faculty of Medicine and Biomedical Sciences, University of Yaoundé I, Yaoundé, Cameroon; 3 Department for the Control of Disease, Epidemics and Pandemics, Ministry of Public Health, Yaoundé, Cameroon; 4 Department of Pediatrics, Douala Laquintinie Hospital, Douala, Cameroon; 5 Department of Epidemiology and Public Health, Centre Pasteur of Cameroon, Rue Henry Dunant; P.O, 1274 Yaoundé, Cameroon; 6 School of Public Health, Faculty of Medicine, University of Paris Sud XI, Le Kremlin Bicêtre, France

**Keywords:** HIV/AIDS, Community dispensation, Sustainability, Mutual, Cameroon

## Abstract

**Background:**

The advent and widespread use of antiretroviral therapy (ART) has remarkably changed the paradigm of HIV infection, increasing substantially the lifespan and quality of life of people affected. Accordingly and responding to policy makers and international directives, many strategies were put in place in Cameroon to accelerate ART uptake, including the community dispensation of ART through community-based organizations (CBOs).

**Main body:**

In its strategic plan to curb the burden of HIV/AIDS and as part of accelerating and reinforcing the provision of ART to all people living with HIV (PLWH), Cameroon opted for different strategies including the dispensation of ART in the community through well identified and tutored CBOs. Actually, financing of ART in Cameroon is mainly the conjugation of resources from the Government and its technical and financial partners, basically the Global Funds supplemented by supports from the Unitaid initiative which allows PLWH residing in Cameroon to benefit from continuous ART without spending a dime. However, this external funding will end-up by 2020. Therefore, there is urgent need to think of alternative and efficient strategies to sustain the fight against HIV/AIDS in Cameroon, especially the provision of ART to patients through community dispensation. Some studies carried out in sub-Saharan African countries have shown that mutual health insurance seems to be a solution with great potential to improve access to quality care, mobilize the necessary funds, improve efficiency of the health sector, and promote dialogue and democratic governance in the health sector along with social and institutional development of the society.

**Conclusions:**

The pooling of associations of PLWH in Cameroon and other countries of sub-Saharan Africa in line with the Bamako Initiative constitutes a promising strategy that would undoubtedly help to offset the withdrawal of funding from external sources, and allow an appropriation of the fight against HIV/AIDS by those concerned at the first place. Nevertheless, other lines of research of financing could be explored in the economic sector.

**Electronic supplementary material:**

The online version of this article (10.1186/s40249-019-0518-8) contains supplementary material, which is available to authorized users.

## Multilingual abstracts

Please see Additional file 1 for translations of the abstract into the five official working languages of the United Nations.

## Background

Infection by the human immunodeficiency virus (HIV) remains one of the most dreadful health threats the world has ever experienced. Recent data indicate for instance that 77.3 million persons have been infected since the beginning of the HIV-era, with 35.4 million related deaths. In 2017 specifically, 36.9 million people were living with the virus, including 1.8 million new infections and 940 000 deaths. Sub-Saharan Africa (SSA) constitutes the epicentre of HIV-infection, where almost 80% of HIV-related morbidity and mortality occur [1].

Cameroon is the second country with the heaviest burden of HIV-infection in Central and Western Africa, after Nigeria [2]. The virus affects all age groups, social strata and ethnic groups; women are more affected as it is the case in other SSA countries. Indeed, the latest Demographic and Health Survey conducted in 2011 pointed the prevalence of HIV-infection at 4.3% in Cameroon, 5.6% among women vs 2.9% among men, with a higher prevalence in urban compared to rural areas [3].

Fortunately, the introduction and widespread use of antiretroviral therapy (ART) have totally changed the paradigm of HIV infection, significantly increasing patients’ life expectancy with a consequential improvement in their quality of life [4]. As part of implementation of the strategic plan to combat HIV/AIDS, Cameroon has ambitioned to increase the 12-month survival of people living with HIV (PLWH) on ART to more than 95%, reaching a target of 302 312 PLWH on ART in 2017, and 381 874 by the end of 2018 [5].

In order to achieve this goal, several strategies have been put in place including: (i) the creation of new HIV-care units throughout the country for the routine follow-up and management of PLWH; (ii) the assignment of new tasks within primary health care facilities relating to HIV care, and (iii) the community-based provision of ART through community-based organizations (CBOs). The ultimate goal of these strategies is to expand and improve the provision of care including ART to beneficiaries, and thus to optimize adherence and retention in care of PLWH [5].

Currently, the acquisition and provision of ART to PLWH free of charge remains under the sole responsibility of the Government of Cameroon and its technical and financial partners, specifically the Global Funds to Fight AIDS, Tuberculosis, and Malaria (Global Funds). However, this partnership with the Global Funds is meant to cease by 2020 [5, 6]. Therefore, one of the biggest challenges for the Government of Cameroon will be to sustain accessibility and subsidization of ART once the Global Funds leaves the country. In the present paper, we review the financing of ART in Cameroon, the community-based provision of ART by CBOs, and suggest a model of community participation as a sustainable strategy to efficiently support the provision of ART through community dispensation at the end of the contract with the Global Funds. This strategy could be implemented not only in Cameroon, but also in other countries with similar realities and future challenges.

## Main text

### Financing access and provision of antiretroviral therapy in Cameroon

The year 2007 was a pivotal year in the accessibility of ART in Cameroon. Indeed, free medication in all approved units of care and treatment centres was launched on May 1st, 2007. Financing of ART in Cameroon is mainly the conjugation of resources from the Government and its technical and financial partners, basically the Global Funds which allows PLWH residing in Cameroon to benefit from continuous ART without spending a dime. The Government contributes around 20% while the Global Funds’ contribution is around 80%, for acquisition of ART in Cameroon [6].

However, this provision of ART free of charge to PLWH is the result of a lengthy process initiated in the early 2000s; at that time, acquisition of ART was the sole responsibility of the patient who had to spend around USD 400–480 monthly [6]. In April 2001, the “Access” initiative contributed to the reduction in prices, thanks to an agreement signed on April 4th 2001 between the Cameroon Ministry of Public Health and representatives of pharmaceutical companies; thereby, prices dropped significantly, ranging between USD 44 and 136 per patient per month [6]. In July 2002, another drop in prices occurred following a second agreement signed with an Indian Laboratory which manufactured generic forms of ART. Additionally, the Global Funds created in 2002 entered into play, which contributed to lowering prices of ART up to USD 14 per month [6]. From 2003, Cameroon benefited from several grands from the Global Funds through “Rounds”, supplemented by supports from the Unitaid initiative, until free-of-charge treatment for all was established in 2007 [6].

### Community dispensation of antiretroviral therapy in Cameroon

In its strategic plan to curb the burden of HIV/AIDS and as part of accelerating and reinforcing the provision of ART to all PLWH, Cameroon opted for different strategies including the dispensation of ART in the community through well identified and tutored CBOs [5]. This strategy was put in place in accordance with the World Health Organization (WHO) and Joint United Nations Programme on HIV/AIDS (UNAIDS) guidelines advocating community involvement in HIV and ART service provision [7, 8]. Initiated in 2016, the model involves the creation of community distribution points for ART; it was first implemented in urban areas where the large majority of patients and treatment centres are located, with cohorts of PLWH varying between 4000 and 15 000 persons per centre.

CBOs constitute the cornerstone for the success of this strategy; accordingly, they should be recruited on the basis of rigorous criteria. Priority is given to associations uniting PLWH and with proven experience in developing and implementing specific activities for the fight against HIV/AIDS. These CBOs are tutored by the nearest HIV-care unit or treatment centre. This mentorship comprises: (i) the supply of ART to CBOs; (ii) the supervision of ART provision to patients; (iii) the coordination of CBOs’ activities, and (iv) the monitoring and evaluation [5]. These CBOs benefit from a financial support from the Global Funds through the National AIDS Control Committee, which permits them to cover all costs induced by the implementation of this activity in their respective communities.

It is expected that this community dispensation of ART will alleviate the heavy workload in HIV-care units and treatment centres with consequential reduction in patients’ waiting time and increase in quality and quantity of care delivered both at the hospital or in CBOs. Additionally, it will gradually increase the geographical accessibility to care by bringing ART closer to patients, especially for those who live in remote areas. Another advantage lies in the fact that patients are offered more flexibility regarding their schedule and availability to visit the CBO and take their drugs. Ultimately, retention in care would be ameliorated and the number of those lost to follow-up significantly diminished [5]. In fact, evidence has demonstrated that community-based ART programs can help to improve HIV continuum of care by increasing the proportion of HIV testing, favouring early initiation of ART, overcoming barriers to retention in care, increasing adherence to ART and HIV viral load suppression, decreasing HIV-related morbidity and mortality, and decongesting health services [9–16].

Despite these numerous advantages, this strategy presents some weaknesses, particularly pertaining to its perpetuation and sustainability. Indeed, the current model relies exclusively on external funding through grants from the Global Funds. With the complete departure of the Global Funds from Cameroon in 2020, it is anticipated that if nothing is done from now, the strategy will die soon. Therefore, it is high time we start thinking of alternative and effective strategies to put in place in order to sustain the fight against HIV/AIDS in Cameroon; local leaders and actors should come into play, as well as these associations of PLWH implicated in the community dispensation of ART. We hereby suggest a model of progressive pooling of these associations of PLWH to support the fight against HIVAIDS in Cameroon, especially the community dispensation of ART.

### Community participation through mutual health organizations of people living with HIV

Community participation in the management of chronic diseases and the provision of drugs is an important lever to develop in order to ease universal access of populations to quality care and at a lower cost. Indeed, patients have to play a pivotal role in the improvement of provision of care to them. Accordingly, community-based associations must have sufficient material, financial, and qualified human resources.

Since the introduction of free access to ART in Cameroon in 2007 and despite the support brought in by technical and financial partners, the country has faced several drug shortages. In this regard, local solutions must be quickly considered to overcome current and future shortages in ART supplies at the national and local levels as various donors will withdraw progressively until 2020. In 2016 for instance, the cost for HIV-related activities through the National AIDS Control Committee was about USD 25047010 with the large majority (almost 99%) of these funds being provided by the Global Funds and the President’s Emergency Plan for Aids Relief (PEPFAR). Part of this funding was allocated to cover costs in relation to the community dispensation of ART strategy. Strikingly, one of the major difficulties during that year was the mobilization of resources to be brought in by the local government [17].

Consequently, it is obvious that if nothing is done from now, the upcoming withdrawal of partners will be disastrous for the management of PLWH. Therefore, thinking of a form of mutual is crucial and urgently warranted to anticipate on the decrease or cessation of these external funding for HIV care and management. In the light of experiences from other countries such as Rwanda [18], the creation of community-based mutual for health is a promising alternative to consider. A mutual is defined as a voluntary and not-for-profit insurance structure that is based on mutual assistance, solidarity, and collective sharing of risk of disease. All members participate actively in the management and operation of the mutual. Most often, the mutual gathers a small number of adherents, targeting especially a specific population that is bound by certain characteristics of solidarity [19].

In the Bamako Initiative, a joint WHO/ United Nations Children’s Fund (UNICEF) Initiative aimed at solving the problems in the financing of primary health care in sub-Saharan Africa was launched in September 1987 at a regional WHO meeting [20, 21]. During this meeting, the director of UNICEF dealt with the severe economic crises facing sub-Saharan Africa, the negative effects of adjustment programs on health, and the reluctance of donors to continue to fund recurrent costs of primary health care programs [22]. Following this speech, the African ministers of health present at the meeting adopted a resolution in which they called for the acceleration of primary health care by defining and implementing self-financing mechanisms at district levels, encouraging social mobilization, and ensuring a regular supply of drugs [22].

Accordingly, the pooling of communities for financial participation in the health system, particularly regarding the care of PLWH and provision of ART is in line with the Bamako Initiative [20–22]. Thus, the establishment of mutual will generate local funding that could constitute a special fund not only for the acquisition of drugs in the long term but also for the functioning of community-based organizations. Additionally, these funds could enable the supply of local drug stocks, operation of associations, payment of allowances to persons in charge of distributing ART and setting up of support groups willing to provide psychosocial assistance to patients. On the other hand, they could make it possible to initiate income-generating activities which will alleviate the burden of follow-up exams for indigent patients. Furthermore, the establishment of these mutual health organizations will encourage the involvement of communities in funding the access to ART, easing access to care for the poorer populations and those of the informal sector, and adapting the mutual model to the rural and urban socio-cultural contexts. In addition, it will strengthen the associative system already in place, reinforcing thereby the independence in the management of collected resources and active involvement of each member of the community; this, in turn, will result in higher expectations in the quality and quantity of rendered services [19, 23, 24].

However and beyond these positive aspects, a number of hindrances limit the implementation of mutual at the local level. First and foremost, the ambient poverty which is characteristic of most Cameroonians will jeopardize considerably the capacity of people to contribute. In fact, it is estimated that 37.5% of the population in Cameroon lives below the poverty threshold [25]. What’s worse, this low level of income may be higher among PLWH considering that the higher burden of HIV is among people with low level of income [26]. Other difficulties include the lack of information and political support, the low level of education especially in rural areas, the lack of funding as well as refusal from banks to grant small loans to these associations. Moreover, the strategy seems to match with very specific geographical, demographic and social situations which might limit the utility of this strategy. Therefore, by acting on these different challenges, it may be possible to develop a mutual system adapted to our context and local realities. Before implementing any mutual, it would be interesting and important to investigate which strategy would suit the best to a particular environment or population. In this regard, conducting community-based qualitative and quantitative studies would be paramount to identify such a strategy [27].

The Government of Rwanda has implemented a universal health insurance model which appears to be one of the best experiences in Africa; hence it could serve as a source of inspiration for other African countries facing the same current and/or future challenges described above. Results from the Rwandan community health system (mutual health insurance) are no more a source of debate. For instance, the morbidity attributable to malaria dropped from 64.7% in 2003 to 11.8% in 2008. Likewise, the rate of women giving birth with assistance of health professionals which was around 28% in 2003 increased up to 66.2% only 4 years later. Broadly, the life expectancy increased from 48 to 52 years in eleven years [23, 24]. These performances were acknowledged by the WHO [28], which demonstrates amply the impact of setting up a well-organized and supported mutual system by the State, boosted by a clear political will.

Similarly, several other examples of establishment of mutual associations in sub-Saharan Africa show that despite the major challenges faced by these organizations, they present many opportunities as in Rwanda where the development of this strategy benefited from favourable institutional and political support, hence encouraging populations to adhere [29]. Some studies carried out in sub-Saharan African countries have shown that mutual health insurance seems to be a solution with great potential to: i) improve access to quality care; ii) mobilize the necessary funds; iii) improve efficiency of the health sector; iv) promote dialogue and democratic governance in the health sector along with social and institutional development of the society [19].

### Towards empowering the community dispensation of antiretroviral therapy for PLWH

Looking back at the lessons learned from mutual success stories from various countries of sub-Saharan Africa whose socio-economic context is close to that of Cameroon, we can hypothesize that Cameroon and similar countries have huge advantages that would inspire this model of success in the establishment and running of a health mutual in the community dispensation of ART. These advantages include: the grouping of all existing and well-organized associations of PLWH in Cameroon, the institutional and multi-sectoral involvement in the fight against HIV/AIDS in Cameroon, and the decentralization and delegation of tasks as a valuable strategy for the management of PLWH [5]. These advantages could be potentiated and capitalized for a progressive community ownership of the fight against HIV/AIDS. As a result, in the short-term, the provision of financial support for the functioning and operation of CBOs dispensing ART would be effective. This may include rental of premises, supply of equipment, payment of allowances for staff in charge of community activities, purchase of communication credit, transport to health facilities for the renewal of drug stocks, and payment of costs of follow-up examinations for indigent patients. In the long term, ART could be entirely purchased to compensate for the cessation of external subsidies and the low contribution of the Government [5].

As illustrative and suggested model, a monthly contribution of USD 1 per patient for each of the 381 874 PLWH expected in 2018 would generate around USD 4582488 at the end of a year. In 2016, the financial support provided to each CBO for its functioning totalled around USD 2835 [30]; taking into account that 63 CBOs were recruited throughout the country [30], the total cost to be allocated for CBOs functioning and operation would be around USD 178605 annually. These projections clearly demonstrate that the financial participation of associations of PLWH will not only ensure the sustainability of the community dispensation strategy but also the extension of the number of associations across the country to bring drugs closer to populations, especially those living in the most remote localities.

However, a major drawback to this model lies in the fact that many of these PLWH might live below the poverty line; hence they might not be in position of covering their monthly contributions. Nevertheless, even if only 50% of these patients fulfil their contributions, there will still be enough funds (USD 2291244) to support the strategy. Furthermore, some of the remaining funds could serve to finance income-generating activities to be initiated by these mutual. The additional funds generated will cover contributions for those clearly identified as being unable to contribute. Moreover, if the monthly contribution is increased up to USD 2–3 per patient, the funds generated would also support other related activities for the fight against HIV/AIDS in the country, such as administrative and/or logistic costs and/or payments for case workers or acquisition of drugs. This additional support to the Government could substantially help to prevent shortages of drugs, and increase quality and quantity of care delivered to PLWH. As a matter of fact, the success of implementing such a care system will be the first step towards progressive appropriation of activities relating to the fight against HIV/AIDS by associations and communities of PLWH. This community financial contribution would constitute a substantial support and increase the Government subsidies dedicated to the fight against HIV/AIDS in order to avoid exclusive reliance on external grants from donors.

## Conclusions

The pooling of associations of PLWH in Cameroon and other countries of sub-Saharan Africa constitutes a promising initiative that would undoubtedly help to offset the withdrawal of funding from external sources, and allow an appropriation of the fight against HIV/AIDS by those concerned at the first place. Nevertheless, other lines of research of financing could be explored in the economic sector through the establishment of additional taxes on certain products such as tobacco or alcohol, establishment of a Finance Law by the Parliament exclusively devoted to the fight against HIV/AIDS and the purchase of antiretroviral drugs.

## Additional file

Additional file 1: Multilingual abstracts in the five official working languages of the United Nations. (PDF 302 kb)

## Abbreviations

AIDS: Acquired immunodeficiency syndrome
ART: Antiretroviral therapy
CBOs: Community based organizations
HIV: Human immunodeficiency virus
PLWH: People living with HIV
WHO: World Health Organization
