# Supplementary material for: Sustaining the community dispensation strategy of HIV antiretroviral through community participation
Source: Infect Dis Poverty. 2019 Jan 24;8:5. doi: 10.1186/s40249-019-0518-8 (PMC6345033; doi:10.1186/s40249-019-0518-8)
Supplement: Supplementary file 1 — Multilingual abstracts in the five official working languages of the United Nations. (PDF 302 kb) [file 40249_2019_518_MOESM1_ESM.pdf]

Translation of the abstract into the five official working languages of the United Nations

الحفاظ على استراتيجية توزيع المجتمع للأدوية المضاد لفيروس نقص المناعة البشرية عن طريق المشاركة المجتمعية

بيبيان سيايو كاميني، جوبريت ريتشي نانسو، ساندرا أيوك تاهت، جان جويل بِيجنا

المعلومات الأساسية:لقد أدى ظهور العلاج المضاد للفيروسات الرجعية وانتشاره على نطاق واسع إلى تغيير ملحوظ في نموذج الإصابة بفيروس نقص المناعة البشرية، مما زاد بشكل كبير من عمر ونوعية حياة الأشخاص المتأثرين. وبناءً على ذلك واستجابة لصانعي السياسات والتوجيهات الدولية، تم وضع العديد من الاستراتيجيات في الكاميرون لتسريع تناول الأدوية المضاد للفيروسات الرجعية بما في ذلك توزيعها عن طريق المنظمات المجتمعية (CBOs).
الجزء الأساسي: في خطتها الاستراتيجية للحد من عبء فيروس نقص المناعة البشرية/ الإيدز وكجزء من تسريع وتدعيم توفير العلاج المضاد للفيروسات الرجعية لجميع المصابين بفيروس نقص المناعة البشرية، اختارت الكاميرون استراتيجيات مختلفة بما في ذلك توزيع العلاج المضاد للفيروسات الرجعية في المجتمع من خلال تحديد و تدرس منظمات المجتمع المحلي.
في الواقع، فإن تمويل العلاج المضاد للفيروسات الرجعية في الكاميرون هو في الأساس اقتران الموارد من الحكومة وشركائها الفنيين والماليين، وبشكل أساسي الصناديق العالمية المكملة بالدعم من مبادرة يونيتيد (UNITAID) التي تسمح للأشخاص المصابين بمرض الإيدز أو نقص المناعة البشرية (PLWH) المقيمين في الكاميرون بالاستفادة من العلاج المضاد للفيروسات الرجعيةالمستمر دون إنفاق عشرة سنتات. ومع ذلك، فإن هذا التمويل الخارجي سينتهي بحلول عام 2020. ولذلك، هناك حاجة ملحة للتفكير في استراتيجيات بديلة وفعالة للحفاظ على مكافحة فيروس نقص المناعة البشرية/ الإيدز في الكاميرون، وخاصة توفير العلاج المضاد للفيروسات الرجعية للمرضى من خلال التوزيع المجتمعي. أظهرت بعض الدراسات التي أجريت في بلدان أفريقيا جنوب الصحراء الكبرى أن التأمين الصحي المتبادل يبدو حلاً ذا إمكانية كبيرة لتحسين الوصول إلى الرعاية الجيدة، وتعبئة الأموال اللازمة، وتحسين كفاءة القطاع الصحي، وتعزيز الحوار والحكم الديمقراطي في القطاع الصحي جنباً إلى جنب مع التنمية الاجتماعية والمؤسسية للمجتمع.
الاستنتاجات: يشكل تجمع رابطات الأشخاص المصابين بمرض الإيدز أو نقص المناعة البشرية (PLWH) في الكاميرون وبلدان أخرى في أفريقيا جنوب الصحراء بما يتماشى مع مبادرة باماكو استراتيجية واعدة من شأنها بلا شك أن تساعد في تعويض سحب التمويل من المصادر الخارجية، والسماح بتخصيص محاربة فيروس نقص المناعة البشرية/ الإيدز من قبل المعنيين في المقام الأول. ومع ذلك، يمكن استكشاف خطوط أخرى من البحث في مجال التمويل في القطاع الاقتصادي.

Translated from English version into Arabic by Mohamed Fouad and Amal Alaboud, through

TRANSLATORS WITHOUT BORDERS

维持 HIV 抗逆转录病毒治疗的社区支持

Bibiane Siaheu Kameni, Jobert Richie Nansseu, Sandra Ayuk Tatah, Jean Joel Bigna

引言：抗逆转录病毒治疗（Antiretroviral therapy，ART）的出现和广泛使用，显著改变了 HIV 的感染模式，大大延长了感染人群的寿命，提高了其生活质量。为响应决策者和国际指令，喀麦隆制定了许多策略，以推广抗逆转录病毒治疗的，如基于社区组织（Community-based organizations，CBOs）的 ART 社区参与策略。
正文：在减轻 HIV/AIDS 负担的战略计划中，为加强对所有艾滋病毒携带者（People living with HIV，PLWH）开展抗逆转录病毒治疗，喀麦隆选择适当的社区组织进行

指导，然后在社区中推广抗逆转录病毒治疗。该项目主要由政府及其技术和财务合作伙伴-全球基金提供资金支持；其次受 UNITAID 倡议支持，居住在喀麦隆的 HIV 感染者能免费接受抗逆转录病毒治疗。然而，2020 后外部资金停止，因此，喀麦隆迫切需要寻找其他有效策略，以继续防治 HIV/AIDS，特别是通过社区管理加强抗逆转录病毒治疗这一策略。部分撒哈拉以南非洲国家的研究表明，共同健康保险似乎能解决该问题，并有望提高获得优质护理的机会，调动必要的资金，提高卫生部门的效率，增强卫生部门的民主对话与治理，以及促进社会体制发展。
结论：在《巴马科倡议》框架下，把喀麦隆和撒哈拉以南非洲国家把艾滋病病毒携带者协会联合起来，将有望弥补外部撤走的资金，并鼓励有关各方首先拨出防治 HIV/AIDS 的款项。另外，经济部门也可以探索其他筹资方法。

Translated from English version into Chinese by Sheng-Lin Wang, edited by Jin Chen

CHINESE CENTER FOR DISEASE CONTROL AND PREVENTION
NATIONAL INSTITUTE OF PARASITIC DISEASES

Soutenir la stratégie de distribution communautaire du traitement antirétroviral à travers la participation des populations

Bibiane Siaheu Kameni, Jobert Richie Nansseu, Sandra Ayuk Tatah, Jean Joel Bigna

Contexte: L’émergence et l’usage répandu du traitement antirétroviral (TARV) ont considérablement modifié le paradigme de traitement de l’infection par le VIH et augmenté considérablement l’espérance de vie et la qualité de vie des personnes infectées. Par conséquent, en réponse aux instructions des décideurs et aux directives internationales, un grand nombre de stratégies ont été mises en place au Cameroun afin d’accélérer la diffusion du TARV, notamment sa distribution communautaire par l’intermédiaire d’organisations communautaires (OC).
Discussion : Dans son plan stratégique pour réduire le fardeau du VIH/SIDA et dans le cadre de l’accélération et du renforcement de l’approvisionnement en TARV de toutes les personnes infectées par le VIH, le Cameroun a opté pour différentes stratégies, notamment la distribution du TARV au sein des communautés par des organisations communautaires bien identifiées et encadrées. Au Cameroun, le TARV est principalement financé par une conjugaison de ressources du gouvernement et de ses partenaires techniques et financiers, essentiellement les fonds mondiaux complétés par le soutien de l’initiative UNITAID qui permet aux personnes séropositives résidant au Cameroun de bénéficier d’un TARV continu sans devoir dépenser un centime. Toutefois, ce financement externe s’arrêtera d’ici 2020. Il est donc urgent de réfléchir à des alternatives et stratégies efficaces à l’appui de la lutte contre le VIH/SIDA au Cameroun, et en particulier de l’approvisionnement en ARV des patients par la distribution communautaire. Certaines études menées dans des pays d’Afrique subsaharienne ont montré qu’une assurance maladie mutuelle semblait être une solution prometteuse pour améliorer l’accès à des soins de qualité, mobiliser les fonds nécessaires, améliorer l’efficacité du secteur de santé et promouvoir un dialogue et une gouvernance démocratiques dans le secteur de la santé parallèlement au développement social et institutionnel de la société.
Conclusions: Le regroupement d’associations de personnes porteuses du VIH au Cameroun et dans d’autres pays d’Afrique subsaharienne en lien avec l’Initiative de Bamako représente une stratégie prometteuse, qui aidera sans aucun doute à compenser la suppression des financements extérieurs.

Il permettrait également aux personnes directement concernées par la lutte contre le VIH/SIDA de s’approprier ce combat. Néanmoins, d’autres sources de financement pourraient être recherchées dans le secteur économique.

Translated from English version into French by Marie Piaget and Suzanne Assenat, through

TRANSLATORS
WITHOUT BORDERS

Обеспечение устойчивости стратегии предоставления антиретровирусной терапии с привлечением общин

Бибиан Сиахеу Камени, Джобберт Ричи Нанссеу, Сандра Аюк Татах, Джин Джоел Бигна

История вопроса: Внедрение и широкое применение антиретровирусной терапии (АРТ) значительно изменило картину ВИЧ: продолжительность жизни инфицированных увеличилась, качество их жизни стало выше. В соответствии с указаниями директивных органов и международных организаций, в Камеруне были реализованы различные стратегии ускоренного распространения АРТ, в частности, за счет предоставления АРТ на уровне общин с привлечением общинных организаций (ОО).
Основная часть: Национальный стратегический план облегчения бремени ВИЧ/СПИДа, принятый Камеруном в русле усилий по ускорению и расширению предоставления АРТ всем инфицированным ВИЧ (ЛЖВ), определил ряд стратегий, одна из которых предполагает предоставление АРТ на уровне общин с привлечением должным образом отобранных и подготовленных ОО. Фактически, финансирование АРТ в Камеруне осуществляется за счет ресурсов государства и его технических и финансовых партнеров, в числе которых, в основном, глобальные фонды. Кроме того, поддержка оказывается по линии инициативы ЮНИТЭЙД. За счет этого ЛЖВ, проживающие в Камеруне, могут непрерывно получать АРТ на бесплатной основе. Однако в 2020 году внешнее финансирование будет прекращено. Это означает, что необходимо продумать эффективные альтернативные стратегии обеспечения устойчивости борьбы с ВИЧ/СПИДом в Камеруне, особенно в части предоставления АРТ на уровне общин. Результаты ряда исследований, проводившихся в странах Африки к югу от Сахары, указывают, что значительным потенциалом в плане расширения доступа к качественным медицинским услугам, повышения эффективности системы здравоохранения, содействия диалогу и демократическому управлению национальным здравоохранением, социального и институционального развития обладает взаимное медицинское страхование.
Заключение: Объединение ассоциаций ЛЖВ в Камеруне и других странах Африки к югу от Сахары в русле инициативы Бамако представляет собой многообещающую стратегию, которая несомненно поможет компенсировать прекращение внешнего финансирования и позволит продолжить борьбу с ВИЧ/СПИДом силами тех, кто в первую очередь заинтересован в победе. Параллельно следует вести в национальной экономике поиск других источников финансирования.

Translated from English version into Russian by Elena McDonnell and Alexander Poddubnyy, through

Sostener la estrategia de dispensación de antirretrovirales para el VIH en la comunidad a través de la participación de esta.

Bibiane Siaheu Kameni, Jobert Richie Nansseu, Sandra Ayuk Tatah, Jean Joel Bigna

Antecedentes: La llegada y el uso generalizado de terapia antirretroviral (TARV) ha cambiado enormemente el paradigma de la infección por VIH, lo que ha aumentado sustancialmente la esperanza y la calidad de vida de las personas afectadas por la enfermedad. Por consiguiente y en respuesta a la legislación y a las directrices internacionales, se han puesto en marcha muchas estrategias en Camerún para acelerar el uso de TARV, incluida la dispensación comunitaria de TARV a través de organizaciones basadas en la comunidad (CBO).
Cuerpo principal: En su plan estratégico para aliviar el lastre que supone el VIH/SIDA y como parte de la aceleración y refuerzo de provisiones de TARV a todas las personas que viven con VIH (PVVIH), Camerún ha optado por diferentes estrategias, incluida la dispensación de TARV en la comunidad a través de CBO bien identificadas y tutorizadas. De hecho, la financiación de TARV en Camerún es el resultado de recursos del Gobierno y de sus Socios Financieros y Técnicos, que son, básicamente, los Fondos Globales complementados por apoyos provenientes de la iniciativa UNITAID, que permiten a las PVVIH que viven en Camerún beneficiarse de una TARV continua sin gastar un solo céntimo. Sin embargo, esta financiación externa finalizará en 2020. Por tanto, existe una necesidad apremiante de pensar en estrategias alternativas y eficientes que mantengan la lucha contra el VIH/SIDA en Camerún, especialmente el aprovisionamiento de TARV a pacientes a través de la dispensación de la comunidad. Algunos estudios llevados a cabo en países del África Subsahariana han mostrado que los seguros de salud mutuos parecen ser una solución con un gran potencial para mejorar el acceso a una atención de calidad, movilizar los fondos necesarios, mejorar la eficiencia del sector de la salud y promocionar el diálogo y la gestión democrática en el sector de la salud junto con el desarrollo social e institucional de la sociedad.
Conclusiones: El conjunto de asociaciones de PVVIH en Camerún y otros países del África Subsahariana junto con la Iniciativa Bamako constituye una estrategia prometedora que ayudaría, sin lugar a dudas, a compensar la retirada de financiación por parte de fuentes externas, y permitiría que aquellos a quienes afecta en primer lugar el VIH/SIDA pudieran luchar contra él. Sin embargo, se podrían explorar otras líneas de financiación en el sector económico.

Translated from English version into Spanish by Tanya Fernández Escudero and Tamcorgil, through

TRANSLATORS
WITHOUT BORDERS
